# Immune-associated molecular occurrence and prognosis predictor of hepatocellular carcinoma: an integrated analysis of GEO datasets

**DOI:** 10.1080/21655979.2021.1962147

**Published:** 2021-08-23

**Authors:** Guanjun Liu, Dongmei Wu, Yiyang Wen, Shundong Cang

**Affiliations:** a Department of Oncology, Henan Provincial People’s Hospital, Zhengzhou, Henan, P.R. China; b Department of Oncology, People’s Hospital of Zhengzhou University, Zhengzhou, Henan, P.R. China; c Department of Oncology, People’s Hospital of Henan University, Zhengzhou, Henan, P.R. China; d Department of Radiotherapy, Xinxiang Center Hospital, Xinxiang, Henan, P.R. China

**Keywords:** Molecular predictor, immune, hepatocellular carcinoma, occurrence, prognosis, cd8^+^ t cells

## Abstract

Hepatocellular carcinoma (HCC) is the fifth most common cancer and the second most common cause of cancer-related deaths worldwide. As immune response failure is the main factor in the occurrence and poor prognosis of HCC, our study aimed to develop an immune-associated molecular occurrence and prognosis predictor (IMOPP) of HCC. To that end, we discovered a 4-gene immune-associated gene signature: C–C motif chemokine ligand 14 (*CCL14*), kallikrein B1 (*KLKB1*), vasoactive intestinal peptide receptor 1 (*VIPR1*), and cluster of differentiation 4 (*CD4*). When tested on three cohorts as an immune-associated molecular occurrence predictor (IMOP), it had high sensitivity, specificity, and area under the receiver operating characteristics curve. When tested as an immune-associated molecular prognosis predictor (IMPP), it stratified the HCC prognosis for overall survival (Kaplan–Meier analysis, log rank *P* = 0.0016; Cox regression, HR = 1.832, 95% CI = 1.173–2.859, *P* = 0.008) and disease-free survival (Kaplan-Meier analysis, log rank *P* = 0.0227). IMPP also significantly correlated with the clinicopathological characteristics of HCC; integrating it with clinicopathological characteristics improved the accuracy of a nomogram for overall survival prediction (C-index: 0.7097 vs. 0.6631). In HCC tumor microenviroments, the proportion of CD8^+^ T cells significantly differed between IMOP-stratified groups. We conclude that IMOPP can potentially predict the occurrence of HCC in high-risk populations and improve prognostic accuracy by providing new biomarkers for risk stratification. In addition, we believe that the IMOP mechanism may be related to its effect on the proportion of CD8^+^ T cells in tumor-infiltrating lymphocytes.

## Graphical abstract

**Figure:**
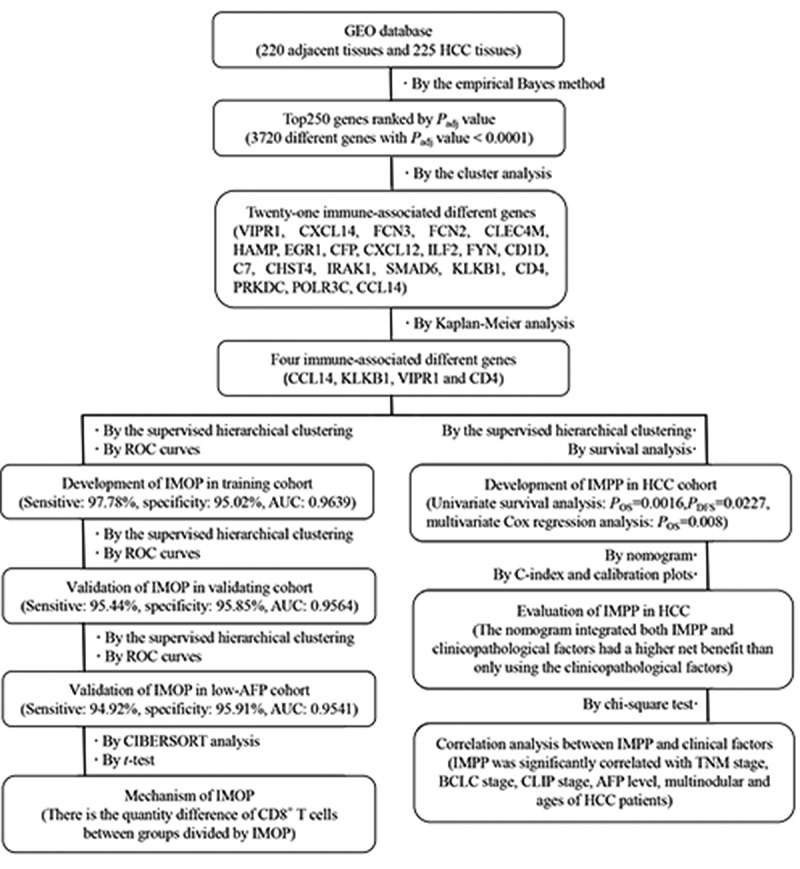

## Introduction

Hepatocellular carcinoma (HCC) is the fifth most common cancer and the second most common cause of global cancer-related deaths [1]. Approximately 782,500 new HCC cases and 745,500 deaths occurred worldwide during 2012, with China alone accounting for roughly 50% of the total number of cases and deaths [2]. More worriesome is the incidence and mortality of HCC, which have been increasing internationally in recent decades [2,3]. However, molecular mechanisms involved in the development of HCC remain ambiguous, and therapeutic methods are limited largely to surgical resection and liver transplantation. Diagnostic delays, high recurrence rates, and high metastatic rates contribute to a 5-year survival rate of less than 30% in HCC patients who undergo surgical resection [4]. As early HCC diagnosis and the identification of HCC patients with poor prognosis are critical to improving survival, investigating the biomarkers for HCC occurrence and prognosis is of considerable importance.

Serum alpha fetal protein (AFP) has been used extensively for decades as a molecular predictor for the diagnosis of HCC. However, its suitability for HCC surveillance is reportedly low [5]. In recent years, several molecular predictors for the occurrence and prognosis of HCC have been proposed [6,7]. Unfortunately, these molecular predictors are not always suitable for evaluating the occurrence and prognosis of HCC [6,7].

Immune response, an important factor in maintaining internal homeostasis, reportedly plays a role in immune surveillance and prevention of infections. Immune-associated genetic dysfunction is the key cause of cancer development and occurrence [8,9]. Ahmad et al. found that Toll-like eceptor 4 can suppress antitumor response and trigger the development of ultraviolt B–induced skin cancer [10]; Nicoud et al. [11] discovered that histamine H_4_ receptor can upregulate the proportion of cluster of differentiation (CD)4^+^ CD25^+^ FoxP3^+^ regulatory T cells and promote an immunosuppressive milieu. Immune-associated predictors have been shown to accurately predict the occurrence or prognosis of ovarian cancer and colorectal cancer [12–14]. For example, a study on Ewing sarcoma [15] reported that a gene signature consisting of 11 immune-associated genes correlates with patient prognosis and can be used as a reliable prognostic biomarker. However, there are few studies applying immune-associated signatures to the prediction of HCC occurrence and prognosis.

In the current study, we hypothesized that immune-associated genes play an important role in HCC development. We aimed to develop an immune-associated molecular occurrence and prognosis predictor (IMOPP) for HCC and evaluate its potential mechanisms. In order to accomplish our goals, we first analyzed data from the Gene Expression Omnibus (GEO) database on 445 patients with HCC to identify differentially expressed immune-associated genes for HCC. We then developed and validated an immune-associated molecular occurrence predictor (IMOP) by using three cohorts that had been established by two independent GEO datasets, and we evaluated the IMOP using areas under the receiver operating characteristic (ROC) curve (AUC). At the same time, we developed an immune-associated molecular prognosis predictor (IMPP), which we evaluated by using survival analysis. In addition, we constructed a nomogram by combining the IMPP with clinicopathological characteristics to increase the accuracy of prognostic predictions. Finally, we used a CIBERSORT algorithm to determine the potential mechanism of IMOP in the development of HCC.

## Methods and materials

### Study design

In this retrospective study of data from two GEO databases, we aimed to develop an IMOPP that can identify patients at high risk of HCC occurrence and those with poor HCC prognosis. First, we reanalyzed the GSE14520 database utilizing the GPL3921 platform (GSE14520-GPL3921) and identified immune-associated genes by means of cluster analysis and Kaplan–Meier analysis. Second, an IMOP was developed and validated by analyzing AUC in a training cohort, validation cohort, and low-AFP cohort. Third, an IMPP was developed using an HCC cohort, and IMPP efficiency was evaluated by Kaplan–Meier analysis, Cox regression, Harrell’s concordance index (C-index), and a calibration plot. Finally, we used the CIBERSORT algorithm to quantify tumor-infiltrating immune cells in tumor microenvironments and discover the potential mechanism of the HCC IMOP.

### Patients and cohorts

For the HCC IMOP, we used data collected from a total of 878 patients in two GEO databases. To develop the IMOP, we established a training cohort using all 445 tissues in the GSE14520-GPL3921 dataset (https://www.ncbi.nlm.nih.gov/geo/query/acc.cgi?acc=GSE14520). To validate the IMOP, we established a validation cohort using all 433 tissues in the GSE36376 dataset (https://www.ncbi.nlm.nih.gov/geo/query/acc.cgi?acc=GSE36376). To establish the low-AFP cohort, we chose 220 adjacent tissues and 118 HCC tissues with AFP levels lower than 300 ng/mL from the GSE14520-GPL3921 dataset. Detailed clinical information and informed consent had been separately provided to and reported by Roesssler S et al. [16,17] and Lim HY [18].

Owing to insufficient survival information, we developed the IMPP by choosing 221 HCC tissues from the GSE14520-GPL3921 dataset to establish an HCC cohort (Supplementary Table 1). The endpoints were HCC overall survival (defined as the time from surgery to death) and HCC disease-free survival (defined as the time from surgery to any recurrence, distant metastasis, or death from any cause) as described by Roesssler and colleagues [16,17].

### Identification of differentially expressed immune-associated genes

In order to identify differentially expressed genes associated with the pathogenesis of HCC, we downloaded the GSE14520-GPL3921 microarray expression profile and reanalyzed it using ‘limma’ package in the R programming environment (v2.15.3) [19]. Then, the top 250 of those genes with *P* values < 0.001 were chosen for further Gene Ontology (GO) analysis using the DAVID website (https://david.ncifcrf.gov/) [20]; we then focused on the pathways associated with immune response (*P* value < 0.05).

### Development and validation of the IMOPP

Machine learning is a type of artificial intelligence that is widely used for the diagnosis and prognosis of disease [6]. In the present study, we used hierarchical clustering to develop and validate an IMOP. We first extracted immune-associated gene expression data from the training cohort, validation cohort, and low-AFP cohort. Then, hierarchical clustering (based on the Manhattan distance and the clustering methods of Ward. D) was conducted using ‘pheatmap’ package in the R programming environment (v3.2.2).

We used the HCC cohort to develop an IMPP, employing the same strategy as that used for IMOP. Generally, all patients were categorized into two groups: the normal immunity group (containing patients with relatively more adjacent tissues or better prognosis) and the immunodeficient group (containing patients with relatively fewer adjacent tissues or poorer prognosis).

### Evaluation of tumor-infiltrating immune cells in tumor microenvironment

CIBERSORT algorithm is an analytical tool that uses gene expression data to evaluate specific cell types within a mixed cell population [21]. To estimate the composition of tumor-infiltrating immune cells in HCC and quantify the relative levels of distinct immune cell types, we utilized the CIBERSORT platform (https://cibersort.stanford.edu/). The analysis was performed using an arrangement of 100 default statistical parameters. At a threshold of *P* < 0.05, results of the inferred fractions of tumor-infiltrating immune cells produced by CIBERSORT were considered accurate. When the average relative percent of tumor-infiltrating immune cells exceeded 0.01 for any group, a comparison was performed between the normal immunity group and the immunodeficient group.

### Statistical analysis

In the R programming environment, ROC curves were plotted with the ‘pROC’ package, and cluster analysis was performed using the ‘GOplot’ package. The chi-square test was used for analyzing the correlation between two groups, Kaplan–Meier method was used to compare overall survival and disease-free survival between different groups, and the log-rank test was used to estimate the significance of differences in survival rates. Survival curves were plotted with Graphpad prism 5.0 software (GraphPad Software Inc., San Diego, CA, USA). Cox regression was utilized to analyze overall survival and disease-free survival, and results were reported as hazard ratios (HR) with 95% confidence intervals (CI). A nomogram combining the risk score model with clinicopathological characteristics was constructed using the ‘rms’ package [22]. Discrimination by the prognostic models was measured and compared using Harrell’s concordance index (C-index) and a calibration plot. Statistical analyses were performed using SPSS Statistics software (v19.0; SPSS Inc., IL, USA). All statistical tests were two-tailed, and *P* ≤ 0.05 was considered statistically significant.

## Results

Considering the harm caused by HCC and the important role that immune response plays in HCC development, our aims were to develop an IMOPP for HCC and to evaluate its potential mechanisms. To that end, this study identified an immune-associated gene signature consisting of four genes: C–C motif chemokine ligand 14 (*CCL14*), kallikrein B1 (*KLKB1*), vasoactive intestinal peptide receptor 1 (*VIPR1*) and *CD4*. We found that when tested as an IMOP on three cohorts, the predictor had high sensitivity, specificity, and AUC. When tested as an IMPP, the predictor could stratify the HCC prognosis for overall survival (based on Kaplan–Meier analysis and Cox regression) and disease-free survival (based on Kaplan–Meier analysis). A nomogram that integrated IMPP with clinicopathological characteristics showed improved accuracy in overall survival prediction compared to a nomogram that used only clinicopathological characteristics. In addition, IMPP results also significantly correlated with the clinicopathological characteristics of HCC. In HCC tumor microenvironments, the difference in number of CD8^+^ T cells was significant among IMOP-stratified groups.

### Identification of a differentially expressed immune-associated gene signature for HCC

**Figure 1.**
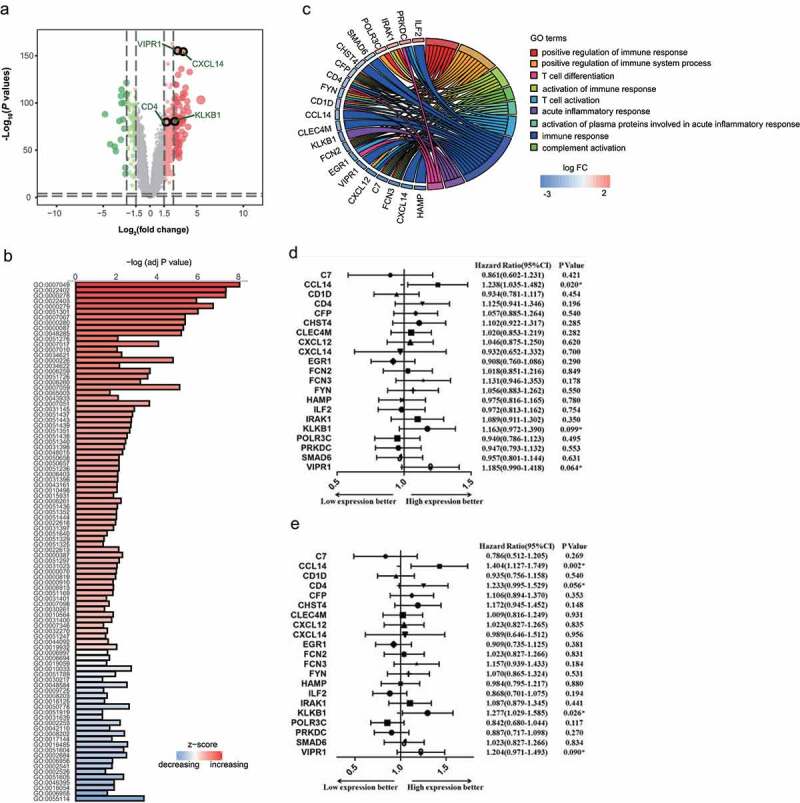
Identification of a differentially expressed immune-associated gene signature for HCC. (a) Volcano plot shows differentially expressed genes based on an absolute log_2_ fold change (FC) >1.5 and adjusted *P* value < 0.05; black circles indicate four immune-associated genes. (b) GO analysis of the top 250 differentially expressed genes. (c) Circos plot illustrating the relationship between differentially expressed immune-associated genes and GO terms for HCC. (d) Univariate analysis of the relationship between differentially expressed immune-associated genes and HCC overall survival. (e) Univariate analysis of the relationship between differentially expressed immune-associated genes and HCC disease-free survival

To gain a global understanding of the pathogenesis of HCC, the GSE14520-GPL3921 microarray expression profile was used to discover genes that were differentially expressed between HCC tissue and adjacent tissues. The preliminary results showed that expression levels significantly differed among 3720 genes (adjusted *P* value < 0.0001, Figure 1(a)). The top 250 of those genes were used for further Gene Ontology (GO) analysis. According to their attributes and biological functions (Figure 1(b)), these differentially expressed genes were classified as: i) cell cycle (e.g., GO: 0007049, GO: 0022402, GO: 0000278), ii) immune response (e.g., GO: 0050778, GO: 0048548, GO:002684), and iii) oxidation reduction (e.g., GO:0055114).

Owing to the importance of their function, special attention was paid to pathways associated with immune response. Twenty-one genes associated with immune response were involved with the immune activation response and acute inflammation response (Figure 1(c)).

Immune-associated genes can influence HCC overall and disease-free survival. Kaplan–Meier analysis revealed that *CCL14* was significantly associated with HCC disease-free survival (Figure 1(d)) and that *CCL14* and *KLKB1* were significantly associated with overall survival (Figure 1(e)). In order to maximize the predictive power of these immune-associated genes, we extended the survival analysis *P* value threshold to 0.1 and thereafter identified four genes for use as an IMOPP of HCC: *CCL14, KLKB1, VIPR1*, and *CD4*.

### Development and validation of an IMOP for HCC

**Figure 2.**
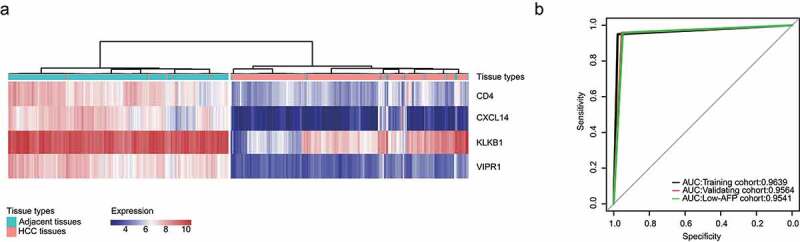
Development and validation of an IMOP for HCC. (a) Development of the IMOP via hierarchical clustering analysis of the training cohort. (b) ROC validation of IMOP in the training cohort, validation cohort, and low-AFP cohort

Clustering, which is widely used for data mining, feature extraction, disease diagnosis and prognostic evaluation, is a fundamental data analysis method in machine learning [23,24]. In order to develop a robust molecular occurrence predictor for HCC, we used a hierarchical clustering algorithm. Results showed that the sensitive and specificity of IMOP in the training cohort were 97.78% and 95.02%, respectively (Figure 2(a), Table 1); the AUC was 0.9639 (Figure 2(b)). For the validation cohort, the sensitive and specificity were 95.44% and 95.85%, respectively (Supplementary Figure 1a, Table 1); the AUC was 0.9564 (Figure 2(b)). For the low-AFP cohort, the sensitive and specificity were 94.92% and 95.91%, respectively (Supplementary Figure 1b, Table 1); the AUC was 0.9541 (Figure 2(b)). High sensitivity, specificity, and AUC can indicate the superiority of a predictor. These results prove that the IMOP possessed significant capability for distinguishing between HCC tissue and adjacent tissues.

**Table 1.** Sensitivity and specificity of the immune-associated molecular occurrence predictor in the training cohort, validation cohort, and low-AFP cohort

Clinical diagnosis	Training cohort			Validating cohort			Low-AFP cohort		
	HCC tissues	adjacent normal tissues	Percent	HCC tissues	adjacent normal tissues	Percent	HCC tissues	adjacent normal tissues	Percent
HCC tissues	220	5	97.78%	230	11	95.44%	112	6	94.92%
adjacent tissues	11	210	95.02%	8	185	95.85%	9	211	95.91%

### Development and evaluation of an IMPP for HCC

**Figure 3.**
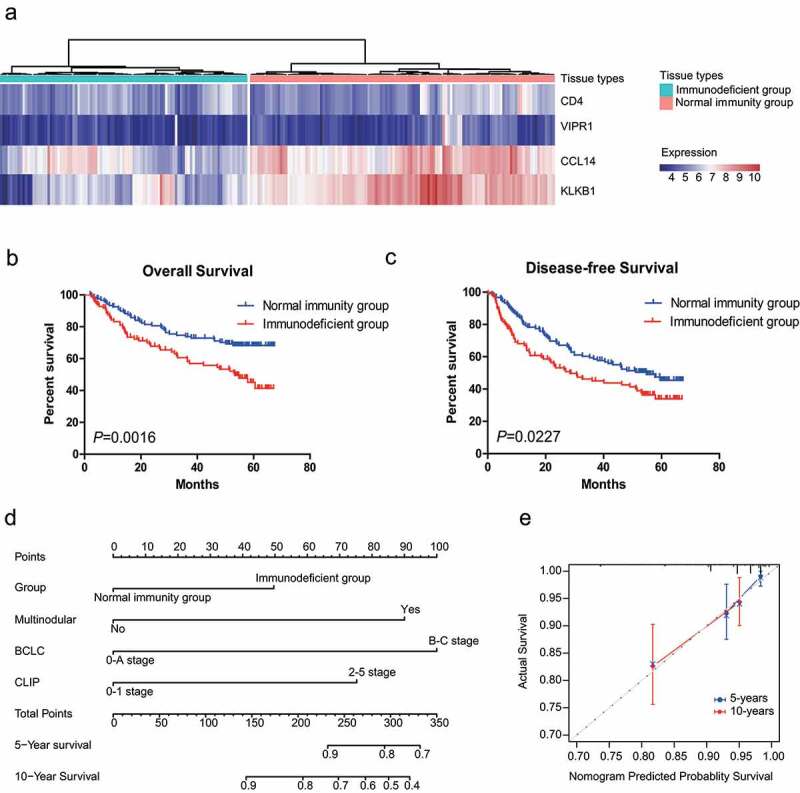
Development and validation of an IMPP for HCC. (a) Development of the IMPP via hierarchical clustering. (b) Overall survival was significantly lower in the immunodeficient group than in the normal immunity group. (c) Disease-free survival was also significantly lower in the immunodeficient group than in the normal immunity group. (d) Nomogram for predicting 5- and 10-year overall survival for HCC patients based on IMPP and clinicopathological characteristics (MN, Multinodular; BCLC, Barcelona Clinic liver cancer; CLIP, Cancer of the Liver Italian Program). (e) Calibration plot illustrating agreement between observed and nomogram-predicted 5- and 10-year outcomes. The 45° dashed line represents perfect prediction; the actual results from our nomogram are indicated by blue and red lines

In the correlation analysis between IMPP and overall survival or disease-free survival in HCC patients, we first used hierarchical clustering to divide HCC patients from the GSE14520-GPL3921 dataset into two groups: the immunodeficient group and the normal immunity group (Figure 3(a)). For prognostic estimation, we found that the IMPP was significantly associated with HCC overall survival (log rank *P* = 0.0016; Figure 3(b)) and disease-free survival (log rank *P* = 0.0227; Figure 3(c)).

In order to eliminate the influence of clinicopathological characteristics on HCC overall survival and disease-free survival, we performed Cox regression analysis on the two IMPP-stratified groups. After eliminating the influence of clinicopathological characteristics, we found that IMPP was significantly associated with HCC overall survival (HR = 1.832, 95% CI = 1.173–2.859, *P* = 0.008; Table 2) but not associated with disease-free survival (*P* = 0.145; Table 2).

**Table 2.** Univariate and multivariate Cox regression analyses of disease-free survival and overall survival between HCC patient groups stratified by the immune-associated molecular prognosis predictor

Characteristics	Univaries analysis		Multivaries analysis	
	Hazard ratio (95% CI)	*P* Value	Hazard ratio (95% CI)	*P* Value
Disease-free survival				
Group (immunodeficient group or normal immunity group)	1.537(1.074–2.200)	0.019	-	0.145
Gender (male or female)	1.480(1.071–2.047)	0.018	1.387(1.001–1.921)	0.049
MTS (≤5 cm or >5 cm)	0.832(0.692–1.001)	0.051	-	0.880
Cirrhosis (no or yes)	-	0.053	-	0.122
TNM stage (I-II stage or III stage)	0.668(0.547–0.816)	0.000	-	0.746
BCLC stage (0-A stage or B-C stage)	0.609(0.500–0.741)	0.000	0.627(0.514–0.764)	0.000
CLIP stage (0–1 stage or 2–5 stage)	0.699(0.573–0.854)	0.000	-	0.754
Overall survival				
Group (immunodeficient group or normal immunity group)	2.029(1.316–3.129)	0.001	1.832(1.173–2.859)	0.008
MTS (≤5 cm or >5 cm)	0.707(0.570–0.877)	0.002	-	0.588
MN (no or yes)	-	0.056	1.729(1.241–2.410)	0.001
Cirrhosis (no or yes)	0.464(0.230–0.935)	0.032	-	0.052
TNM stage (I-II stage or III stage)	0.535(0.428–0.671)	0.000	-	0.119
BCLC stage (0-A stage or B-C stage)	0.533(0.426–0.666)	0.000	0.540(0.380–0.767)	0.001
CLIP stage (0–1 stage or 2–5 stage)	0.561(0.449–0.701)	0.000	0.628(0.444–0.889)	0.009

MTS, Main tumor size; MN, Multinodular; BCLC, Barcelona Clinic liver cancer; CLIP, Cancer of the Liver Italian Program.

To develop a quantitative method for predicting overall survival in HCC patients, we created a nomogram that integrated IMPP with clinicopathological characteristics (Figure 3(d)). Subsequently, we utilized C-index and calibration plots to evaluate the nomogram. C-index analysis revealed that the net benefit was higher for the nomogram that integrated IMPP with clinicopathological characteristics (C-index = 0.7097) than for the nomogram that was constructed using only clinicopathological characteristics (C-index = 0.6631). Similarly, the calibration plot (Figure 3(e)) showed that the nomogram that integrated IMPP with clinicopathological characteristics performed well when compared to the performance of an ideal model.

HCC prognosis is closely related to clinicopathological characteristics such as AFP level, tumor-node-metastasis (TNM) stages, and Barcelona Clinic Liver Cancer (BCLC) stages. For the correlation analysis between IMPP and the clinicopathological characteristics of HCC, we evaluated differences between the immunodeficient group and the normal immunity group using the chi-square test. Our findings showed that IMPP significantly correlated with TNM stage, BCLC stage, Cancer of the Liver Italian Program (CLIP) stage, AFP level, multinodular presentation, and HCC patient age (Table 3).

**Table 3.** Correlation analysis between immune-associated molecular prognosis predictor and the clinicopathological characteristics of HCC patients

Characteristics		immunodeficient group	normal immunity group	Χ^2^	*P* Value
Gender	Male	90(85.6)	101(105.4)	3.073	0.080
	Femal	9(13.4)	21(16.6)		
Age(years)	≤60	87(81.1)	94(99.9)	4.324	0.038^▲^
	>60	12(17.9)	28(22.1)		
HBV virtal status	AVR-CC	27(24.8)	29(31.2)	0.463	0.496
	CC	67(69.2)	89(86.8)		
ALT	≤50 U/L	53(58.2)	77(71.8)	2.071	0.150
	>50 U/L	46(40.8)	45(50.2)		
AFP	≤300 ng/ml	40(53.0)	78(65.0)	12.707	<0.001^▲^
	>300 ng/ml	58(45.0)	42(55.0)		
Main tumor size	≤5 cm	61(63.0)	79(77.0)	0.317	0.573
	>5 cm	38(36.0)	42(44.0)		
Multinodular	no	73(78.8)	103(97.2)	3.851	0.050^▲^
	yes	26(20.2)	19(24.8)		
Cirrhosis	no	5(8.9)	13(11.1)	3.348	0.067
	yes	94(90.1)	109(102.9)		
TNM stage	I-II stage	68(76.1)	102(93.9)	6.930	0.008^▲^
	III stage	30(21.9)	19(27.1)		
BCLC stage	0-A stage	68(75.2)	100(92.8)	5.327	0.021^▲^
	B-C stage	30(22.8)	21(28.2)		
CLIP stage	0–1 stage	69(76.5)	102(94.5)	6.104	0.013^▲^
	2–5 stage	29(21.5)	19(26.5)		

NA, not available; HBV, Hepatitis B Virus; AVR-CC, active viral replication chronic carrier; CC, chronic carrier; ALT, alanine aminotransferase; AFP, Alpha Fetal Protein; BCLC, Barcelona Clinic liver cancer; CLIP,Cancer of the Liver Italian Program.

▲ There are statistically significant between the two groups.

### Analysis of differences in proportion of CD8^+^ T cells between the HCC IMOP–stratified immunodeficient and normal immunity groups

**Figure 4.**
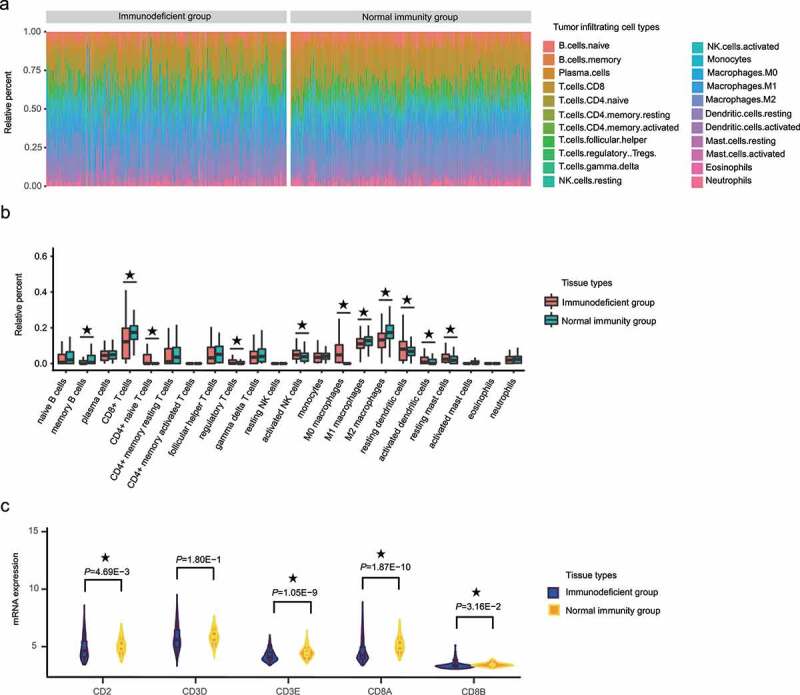
The proportion of CD8^+^ T cells significantly differed between the HCC IMOP–stratified immunodeficient and normal immunity groups. (a) Stacked bar chart showing the relative distribution immune cells (22 cell types) in each sample. (b) Box plot illustrates relative percent of tumor-infiltrating immune cells between the HCC IMOP–stratified immunodeficient and normal immunity groups. (c) Violin plot shows differential expression of CD8^+^ T-cell markers between the HCC IMOP–stratified immunodeficient and normal immunity groups

We used the CIBERSORT algorithm to evaluate the relationship between HCC IMOP and tumor-infiltrating immune cells [21]. As shown in Figure 4(a), the composition of immunity cells (22 cell types) varied significantly among samples; furthermore, a large proportion of the cells were CD8^+^ T cells, CD4^+^ memory resting T cells, follicular helper T cells, gamma delta T cells, activated natural killer cells, M1 macrophages, M2 macrophages, and resting dendritic cells. Mean proportional values for cell types that had a proportion greater than 0.01 in at least one group were compared between immunodeficient and normal immunity groups. The results showed that the memory B cell, CD8^+^ T cell, M1 macrophage, and M2 macrophage ratios were significantly higher in the normal immunity group than in the immunodeficiency group (*P* = 6.58E-4, *P* = 3.65E-10, *P* = 3.43E-4, and *P* = 3.03E-12, respectively; Figure 4(b)). In contrast, the CD4^+^ naive T cell, regulatory T cell, activated natural killer cell, M0 macrophage, resting dendritic cell, activated dendritic cell, and resting mast cell ratios were significantly lower in the normal immunity group than in the immunodeficient group (*P* = 2.21E-8, *P* = 3.42E-2, *P* = 6.91E-3, *P* = 5.18E-24, *P* = 1.80E-3, *P* = 3.27E-5, and *P* = 4.58E-4, respectively; Figure 4(b)). These results indicated that the activation and inhibition of various immune cells in tumor microenvironments occurred simultaneously.

CD8^+^ T cells can affect the development of HCC [25,26]. Thus, we compared the CD8^+^ T-cell markers between immunodeficient and normal immunity groups. The results (Figure 4(c)) showed that the levels of general T-cell markers (CD2 and CD3E) and specific CD8^+^ T-cells markers (CD8A and CD8B) were significantly lower in the immunodeficient group than in the normal immunity group.

## Discussion

Effective methods are needed for predicting HCC occurrence and prognosis in patients. To address this issue, we identified a potential signature involving four immune-associated genes that were differentially expressed between HCC tissue and normal tissues and therefore could be valuable for predicting HCC occurrence and prognosis. Subsequently, we found that the IMOP possessed significant capability for distinguishing between HCC tissue and adjacent tissues in the training cohort, validation cohort, and low-AFP cohort. Meanwhile, the IMPP also showed excellent discriminatory ability in HCC patients with poor prognosis. To develop a quantitative method for predicting the overall survival of HCC patients, we combined clinicopathological characteristics with IMPP to construct a nomogram and then confirmed its performance using C-index and a calibration curve. With regard to the mechanism of IMOP, we found that the difference in proprtion of CD8^+^ T cells was significant among IMOP-stratified groups.

Liver biopsy and pathological diagnosis continue to be the ‘gold standard’ for evaluating chronic hepatitis and fibrosis and for diagnosing HCC [27]. However, liver biopsy and pathological diagnosis have some disadvantages [27,28]. High-risk patients are usually diagnosed with HCC at the intermediate–advanced stage [29]. In tumors, molecular changes often preceed morphological changes [30]. Thus, molecular predictors may be one of the most effective methods for monitoring HCC.

The role of immune response in the occurrence and development of HCC is well recognized [31–33]. Among HCC patients infected by hepatitis B virus (HBV) or hepatitis C virus, immune suppression owing to viral infection and viral gene integration are possible mechanisms associated with the occurrence of HCC [34–36]. The abnormal expression of various characteristic tumor surface antigens can inhibit antitumor immunity, thereby affecting HCC development and HCC treatment [37,38]. The present study found that in HCC patients, *CCL14* was significantly associated with overall survival and disease-free survival, and *KLKB1* was significantly associated with overall survival. Our results concur with those of other studies. In one HCC study, researchers found that *CCL14* can serve as a novel HCC tumor suppressor by regulating cell cycles and promoting apoptosis [39]. Another study on epithelial ovarian cancer revealed that *CCL14* upregulation is associated with a favorable prognosis [40].The differentially expressed immune-associated genes may play important roles in the development of tumor. In order to maximize the predictive power of differentially expressed immune-associated genes, we ensured that the four genes (*CCL14, KLKB1, VIPR1*, and *CD4*) chosen for development as an IMOPP for HCC had *P* values of less than 0.1 for overall or disease-free survival.

Serological tests performed alone or in combination (e.g., AFP, des-gamma-carboxy prothrombin, and AFP-L3) are the main methods for detecting HCC. However, AFP (at a cutoff of 20 ng/mL) has a sensitivity of 40–65% and 14–40% for clinically diagnosed HCC and preclinical HCC, respectively [41]. In addition, the sensitivity and specificity of des-gamma-carboxy prothrombin for clinically diagnosing HCC are only 28–89% and 87–96%, respectively – values which are similar to those for AFP-L3 [41]. Molecular predictors display superior stratification capability. For example, Lin et al. found that one miRNA classifier had high sensitivity (range 70.4–85.7%) and specificity (80.0–91.1%) [6]. In this study, our IMOP had 95% sensitivity and specificity in the training and validation cohorts. That is, our IMOP had higher sensitivity and specificity than do AFP [41,42] and other molecular predictors [6]. Moreover, the sensitivity and specificity of the IMOP reach approximately 95% in the low-AFP cohort. These results show that IMOP has robust capability for distinguishing HCC tissue from adjacent tissue.

Partial hepatectomy is regarded as a standard curative treatment for HCC, but the prognosis for HCC patients who undergo hepatectomy varies even among early-stage HCC patients [43,44]. Therefore, it is important to stratify HCC patients according to pathological parameters, protein biomarkers, mRNA expression levels, and genomic DNA abnormalities [45–47]. In the analysis of prognostic ability, we found that IMPP could effectively be used to divide HCC patients into two groups – the immunodeficient group and the normal immunity group – the latter of which was significantly associated with HCC overall survival and disease-free survival. Even after eliminating the influence of clinicopathological characteristics, IMPP was significantly associated with overall survival. An analysis using C-index and a calibration plot with well-fitted calibration curves showed that a nomogram that integrated IMPP with clinicopathological characteristics had higher accuracy in overall survival prediction (C-index: 0.7097 vs. 0.6631) provided a greater net benefit than than did a nomogram that used only clinicopathological characteristics. In the correlation analysis of IMPP and clinicopathological characteristics, results showed significant differences in TNM stage, BCLC stage, CLIP stage, AFP level, and ages between the two groups of HCC patients. To some extent, IMPP demonstrated its ability to stratify HCC patients with poor prognosis.

The genes associated with immune response are involved in positive regulation of the immune system process, such asT-cell activation and T-cell differentiation. We speculated that a strong immune-associated molecular predictor presence is significantly associated with tumor-infiltrating immune cells. CIBERSORT analysis revealed that the normal immunity group had a higher proportion of CD8^+^ T cells and higher expression levels of CD8^+^ T-cell markers than did the immunodeficient group. As the most predominant tumor-infiltrating lymphocyte, CD8^+^ T cell is ascribed the role of cytotoxic killer cell in cancer immunobiology, and infiltration of CD8^+^ T cell has been regarded as a marker of superior prognosis [48]. In a recent meta-analysis of 3509 patients from 21 observational studies, high levels of intratumoral CD8^+^ T cells were positively correlated with overall survival and disease-free survival [49]. Our experimental results confirmed these results. Regarding cause, most mechanisms of the molecular predictor are obscure in terms of number of CD8^+^ T cells affected. In future studies, we plan to explore the mechanism by which IMOP influences the occurrence of HCC.

There were some limitations in our study. First, we retrospectively studied genetic and clinical data from two independent cohorts in the GEO database; the patients were enrolled in East Asia and had different ethnic and environmental backgrounds. Second, our IMOPP may be limited to HBV-related HCC because the majority of patients in our study were HBV-positive. Owing to these limitations, it will be necessary to validate our predictor in prospective cohorts with different etiological backgrounds and different ethnic characteristics.

## Conclusions

In summary, we developed and validated an IMOPP that can potentially predict the occurrence of HCC in high-risk populations and provide new biomarkers for risk stratification and accurate prognostication. In addition, we found that the IMOP mechanism may involve inducing changes in the number of CD8^+^ T cells. Early diagnosis and accurate stratification of HCC patients help clinicians personalize clinical treatment. Thus, we believe that in spite of its limitations, our HCC IMOPP is important because it allows for individualized treatment recommendations.

## Supplementary Material

Supplemental Material

## Data Availability

Data analyzed during the present study are available at NCBI GEO: GSE14520 and GSE36376.

## Highlights

1. We identified a 4-gene signature for HCC: *CCL14, KLKB1, VIPR1* and *CD4*.
2. IMOPP potentially predicts HCC occurrence and improves prognostic accuracy.
3. IMOP may affect the proportion of CD8^+^ T cells in tumor-infiltrating lymphocytes.
