# Supplementary material for: Immune-associated molecular occurrence and prognosis predictor of hepatocellular carcinoma: an integrated analysis of GEO datasets
Source: Bioengineered. 2021 Aug 23;12(1):5253–65. doi: 10.1080/21655979.2021.1962147 (PMC8806587; doi:10.1080/21655979.2021.1962147)
Supplement: Supplemental Material [file KBIE_A_1962147_SM7289.zip › supplementary/Supplementary Table 1.docx]

| Clinical diagnosis | Training cohort | | |  | Validating cohort | | |  | Low-AFP cohort | | |
| --- | --- | --- | --- | --- | --- | --- | --- | --- | --- | --- | --- |
|  | HCC tissues | adjacent normal tissues | Percent |  | HCC tissues | adjacent normal tissues | Percent |  | HCC tissues | adjacent normal tissues | Percent |
| HCC tissues | 220 | 5 | 97.78% |  | 230 | 11 | 95.44% |  | 112 | 6 | 94.92% |
| adjacent tissues | 11 | 210 | 95.02% |  | 8 | 185 | 95.85% |  | 9 | 211 | 95.91% |
